# Myocardial Gene Expression of *T-bet*, *GATA-3*, *Ror-*γ*t*, *FoxP3*, and Hallmark Cytokines in Chronic Chagas Disease Cardiomyopathy: An Essentially Unopposed T_H_1-Type Response

**DOI:** 10.1155/2014/914326

**Published:** 2014-07-24

**Authors:** Luciana Gabriel Nogueira, Ronaldo Honorato Barros Santos, Alfredo Inácio Fiorelli, Eliane Conti Mairena, Luiz Alberto Benvenuti, Edimar Alcides Bocchi, Noedir Antonio Stolf, Jorge Kalil, Edecio Cunha-Neto

**Affiliations:** ^1^Laboratory of Immunology, Heart Institute (InCor), University of São Paulo School of Medicine, 05403-000 São Paulo, SP, Brazil; ^2^Division of Clinical Immunology and Allergy, University of São Paulo School of Medicine, 01246-903 São Paulo, SP, Brazil; ^3^Institute for Investigation in Immunology (iii), INCT, University of São Paulo School of Medicine, 05403-000 São Paulo, SP, Brazil; ^4^Division of Surgery, Heart Institute (InCor), University of São Paulo School of Medicine, 05403-000 São Paulo, SP, Brazil; ^5^Division of Pathology, Heart Institute (InCor), University of São Paulo School of Medicine, 05403-000 São Paulo, SP, Brazil; ^6^Transplantation and Heart Failure Unit, Heart Institute (InCor), University of São Paulo School of Medicine, 05403-000 São Paulo, SP, Brazil

## Abstract

*Background*. Chronic Chagas disease cardiomyopathy (CCC), a late consequence of *Trypanosoma cruzi* infection, is an inflammatory cardiomyopathy with prognosis worse than those of noninflammatory etiology (NIC). Although the T cell-rich myocarditis is known to play a pathogenetic role, the relative contribution of each of the functional T cell subsets has never been thoroughly investigated. We therefore assessed gene expression of cytokines and transcription factors involved in differentiation and effector function of each functional T cell subset (T_H_1/T_H_2/T_H_17/Treg) in CCC, NIC, and heart donor myocardial samples. *Methods and Results*. Quantitative PCR showed markedly upregulated expression of *IFN*-*γ* and transcription factor *T-bet*, and minor increases of *GATA-3*; *FoxP3* and *CTLA-4*; *IL-17* and *IL-18* in CCC as compared with NIC samples. Conversely, cytokines expressed by T_H_2 cells (*IL-4*, *IL-5*, and *IL-13*) or associated with Treg (*TGF*-*β* and *IL-10*) were not upregulated in CCC myocardium. Expression of T_H_1-related genes such as *T-bet*, *IFN*-*γ*, and *IL-18* correlated with ventricular dilation, *FoxP3*, and *CTLA-4*. *Conclusions*. Results are consistent with a strong local T_H_1-mediated response in most samples, possibly associated with pathological myocardial remodeling, and a proportionally smaller FoxP3^+^CTLA4^+^ Treg cell population, which is unable to completely curb IFN-*γ* production in CCC myocardium, therefore fueling inflammation.

## 1. Introduction

Approximately 8 million people are infected with the protozoan parasite* Trypanosoma cruzi* [1] in Central and South America, with an estimated 300,000 cases in the USA alone due to migration.* T. cruzi* is a major cause of heart disease and cardiovascular-related deaths in endemic areas located in Latin America, with approximately 50,000 fatalities per year due to chronic Chagas cardiomyopathy (CCC) [2]. CCC, the most important clinical consequence of Chagas disease, is an inflammatory cardiomyopathy that affects around 30% of infected individuals and occurs 5–30 years after acute infection, while ca. 60% of those infected remain asymptomatic (ASY) [3]. The reasons why it takes so long after infection for development of full-blown CCC are still unknown. One-third of patients developing CCC present a particularly lethal form of dilated cardiomyopathy with significant left ventricular dysfunction, and shorter survival than cardiomyopathies of noninflammatory etiology (NIC) [4]. CCC is characterized by a diffuse mononuclear cell myocarditis, with significant heart fiber damage, prominent fibrosis, and scarcity of* T. cruzi *parasites (reviewed in [5]). The inflammatory infiltrate of CCC heart lesions is mainly composed by CD4+ and CD8+ T cells and macrophages [6, 7]. The occurrence of myocarditis is correlated with clinical severity, ASY patients having minimal inflammation [8]. Evidence suggests that the presence or intensity of myocarditis plays a major pathogenic role in CCC development and severity.

The immune response to* T. cruzi* is triggered by persistent infection with an obligatory intracellular parasite. During acute* T. cruzi* infection,* T. cruzi* pathogen-associated molecular patterns (PAMPs) trigger innate immunity in multiple cell types [9], which release proinflammatory cytokines, such as IL-1, IL-6, IL-12, IL-18, and TNF-*α*, activating cascades of inflammatory cells [10] (reviewed [11]). Antigen-presenting cells subsequently elicit a strong T cell and antibody response against* T. cruzi*,where IL-12 and IL-18 drive the differentiation of IFN-*γ*-producing* T. cruzi*-specific T_H_1-type T cells which migrate to sites of* T. cruzi*-induced inflammation, including the myocardium, in response to locally produced chemokines [11–13]. The T_H_1-type T cell and antibody responses lead to control—but not complete elimination—of tissue and blood parasitism, establishing a low-grade chronic persistent infection by* T. cruzi*.

As a result of persistent infection, both CCC and ASY chronic Chagas disease patients show a skewed T_H_1-type immune response [13–15] with reduced production of IL-4 by PBMC, but those who develop Chagas cardiomyopathy display a particularly strong T_H_1-type immune response with increased numbers of IFN-*γ*-producing T cells in peripheral blood mononuclear cells (PBMC) [16–18] as well as plasma TNF-*α* in comparison with uninfected or ASY patients [14, 19].

In addition, CCC patients display a reduced number of CD4^+^CD25^high^IL-10^+^ T cells and CD4^+^CD25^high^FoxP3^+^ regulatory T cells in their peripheral blood as compared to patients in the ASY form of Chagas disease, suggesting that such cells may play a role in the control of the intensity of inflammation in chronic Chagas disease [14, 20, 21]. Furthermore, PBMC from CCC patients displayed increased numbers of CD4^+^CD25^high^FoxP3^+^CTLA-4^+^ T cells and decreased numbers of CD4^+^CD25^high^IL-10^+^ T cells as compared to ASY patients. These reports suggest that a smaller CD4^+^CD25^+^ Treg compartment displays a deficient suppressive activity in CCC patients, leading to uncontrolled production of T_H_1 cytokines [22]. Regarding T_H_17 cells in Chagas disease, a recent study showed a lower frequency of circulating CD4^+^IL-17^+^ T cells in CCC patients as compared with ASY patients and noninfected individuals [23].

The exacerbated T_H_1 response observed in the PBMC of CCC patients is reflected on the CD4^+^ and CD8^+^T_H_1-type T cell-rich myocardial inflammatory infiltrate, with mononuclear cells predominantly producing IFN-*γ* and TNF-*α*, with lower production of IL-4, IL-6, IL-7, and IL-15 [6, 7, 14, 16, 24, 25]. It has recently been shown by our group that CCL5^+^, CXCL9^+^, CCR5^+^, and CXCR3^+^ mononuclear cells were abundant in CCC myocardium, and mRNA levels of the T_H_1-chemoattracting chemokines CXCL9, CXCL10, CCL3, CCL4, and CCL5 and their receptors were also found to be upregulated in CCC heart tissue [26]. Significantly, the intensity of the myocardial infiltrate was positively correlated with CXCL9 mRNA expression; moreover, a single nucleotide polymorphism in the* CXCL9* gene, associated with a reduced risk of developing severe CCC in a cohort study, was associated with reduced* CXCL9* expression and intensity of myocarditis in CCC [26]. These results are consistent with a major role of locally produced T_H_1-chemoattractant chemokines in the accumulation of CXCR3/CCR5^+^T_H_1 T cells in CCC heart tissue. Significantly, CCC patients display increased numbers of* T. cruzi*-specific CXCR3^+^ and CCR5^+^ T cells coexpressing IFN-*γ* in the PBMC as compared to ASY subjects [27].

Although the presence of heart-infiltrating T_H_1-type T cells has been well documented, relatively little is known about the presence or relative proportion of the other functional T cell subsets in CCC heart tissue, which may ultimately determine the local inflammatory status. Although studies with PBMC have established significant differences in the frequency of functional T cell subset differences between CCC and ASY, it does not necessarily follow that those findings will all apply to CCC heart tissue. The presence of different Treg populations in CCC heart tissue has been suggested by the findings of Foxp3 expression and TGF-*β* signaling (through Smad4 detection) in CCC compared to ASY heart tissue [28, 29]. Regarding production of IL-4 in CCC myocardium, there are conflicting results, where IL-4-producing mononuclear cells were either undetectable [14], prominent in autopsy samples [25], or outnumbered by IFN-*γ*-producing T cells [30]. So far, T_H_17 cells have not yet been studied in human CCC myocardium.

We believe the elucidation of the balance of functional T cell lineages in CCC myocardium is of paramount importance to understand the pathogenesis of CCC, including the key elements for disease progression.

In order to evaluate the relative contribution of each functional T cell subset in the CCC myocardial inflammatory infiltrate, we assessed the mRNA expression of lineage-specifying transcription factors associated with differentiated T_H_1/T_H_2/T_H_17 T cells (T-box expressed in T cells (T-bet), GATA-binding protein-3 (GATA-3), and retinoid-related orphan receptor *γ*t (ROR*γ*-T), respectively [31, 32] and the corresponding effector cytokines (IFN*γ*, IL-4, IL-5, IL-13, IL-17, and IL-23), along with genes associated with regulatory T cell function (FoxP3, TGF-*β*, CTLA-4, and IL-10), and proinflammatory and/or T_H_1-inducing cytokines (IL-1, IL-6, IL-12p35, IL12p40, IL-18, and IL-23) in myocardial samples from CCC and NIC patients as well as heart donor controls.

## 2. Methods

### 2.1. Ethics Statement

The protocol was approved by the Institutional Review Board of the School of Medicine, University of São Paulo (Protocol number 739/2005) and written informed consent was obtained from the patients. In the case of samples from heart donors, written informed consent was obtained from their families.

### 2.2. Patients and Sample Collection

All Chagas disease patients were considered serologically positive for antibodies against* T. cruzi *on the basis of results of at least 2 of 3 independent tests as described [18]. All Chagas disease and NIC patients underwent standard electrocardiography and 2-dimension and M-mode echocardiography in the hospital setting as described [18]. Patients with CCC presented with typical electrocardiographic findings such as right bundle branch block and/or left anterior division hemiblock [33], in addition to ventricular dysfunction classified on the basis of left ventricular ejection fraction <40%. Myocardial left ventricular free wall heart samples were obtained from end-stage heart failure CCC patients (Table 1) and end-stage heart failure patients with noninflammatory cardiomyopathies (NIC, five patients with idiopathic dilated cardiomyopathy and three patients with ischemic cardiomyopathy, all seronegative for* T. cruzi;* Table 1). Control adult heart tissue from the left ventricular-free wall was obtained from nonfailing donor hearts (N, Table 1) not used for cardiac transplantation due to size mismatch with available recipients. This sample set is the same previously studied for myocardial chemokine expression [26]. Hearts were explanted at the time of heart transplantation at the Heart Institute-InCor, School of Medicine, University of São Paulo, São Paulo, SP, Brazil. For mRNA extraction, samples were quickly dissected, and myocardial tissue was frozen in liquid nitrogen and stored at −80°C.

### 2.3. RNA Isolation, Reverse Transcription, and Quantitative Real-Time Polymerase Chain Reaction (Real-Time qPCR)

Total RNA was extracted from 5 × 5 × 5 mm myocardial samples using the Trizol method (Life Technologies Inc., Grand Island, NY). The RNA was quantified using NanoDrop Spectrophotometry (Thermo Scientific) and treated with Rnase-free DNase I (USB, Ohio, USA). cDNA was obtained from 5 *μ*g total RNA using Super-script II Reverse Transcriptase (Invitrogen, Carlsbad, CA, USA). We designed forward and reverse primers for real-time qPCR assays using the Primer Express software (Applied Biosystems, Foster City, CA, USA; see Table S1 in supplementary materials available online at http://dx.doi.org/10.1155/2014/914326). Real-time qPCR reactions were carried out in an ABI Prism 7500 Sequence Detection system (Applied Biosystems) using the SYBR Green PCR Master Mix (Applied Biosystems), as described in [6]. PCR efficiency was measured in myocardial tissue for all real-time PCR primers. All the samples were tested in triplicate with the glyceraldehyde 3-phosphate dehydrogenase (GAPDH, reference gene) whose expression was previously shown to display little variance among human myocardial tissue samples [24], as the reference gene for normalization of data, and relative expression of each mRNA was calculated with the 2^−ΔΔCt^ method [34], using expression in six normal donor hearts as calibrator. A ratio between expression values of the* T-bet* and* GATA-3* genes was calculated as previously reported [32].

### 2.4. Statistical Analysis

Values of the relative expression of each mRNA in the CCC and NIC groups were compared with the Mann-Whitney *U* test and performed using the GraphPad Prism 5 software. Correlation analysis was performed by Spearman's rank correlation test with SPSS version 14.0 software (SPSS, Chicago, III).

## 3. Results

### 3.1. Patient and Sample Features

As previously observed with the same sample set studied here [26], while myocardial sections from both cardiomyopathy groups displayed cardiomyocyte hypertrophy and fibrosis upon histopathological analysis, lymphocytic myocarditis was only observed among samples from CCC patients (Table 1). No significant differences were found in age, ejection fraction (EF), or left ventricular diastolic diameter (LVDD) between the two groups. We have also previously observed positive correlations between the intensity of lymphocytic myocarditis and fibrosis and between EF and myocardial expression of* ANP* and* BNP* [26]. Myocardial tissue samples are rich in CD4+ and CD8+ T cells (photograph in [26]).

### 3.2. Expression of T_H_1, T_H_2, and T_H_17 T Cell Lineage-Specific of Transcription Factors on Heart Tissue from CCC Patients

**Figure 1:**
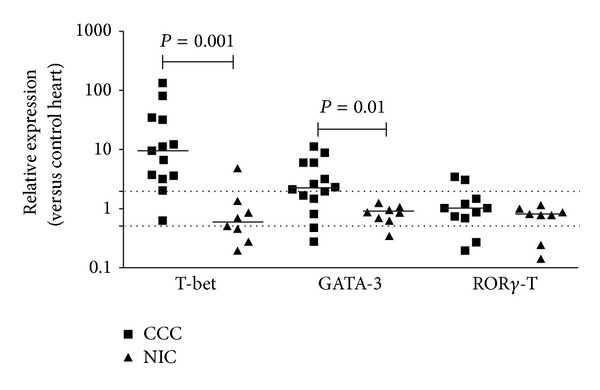
Expression of mRNA encoding transcription factors T-bet, Gata-3, and Ror*γ*-T in myocardium. Real-time qPCR analysis of mRNA expression in CCC and NIC myocardium. After normalization to GAPDH mRNA, relative increase was plotted in comparison to N group and data were calculated with the 2^−ΔΔCt^ method, as described in Methods section. The horizontal bar stands for the median; dotted lines indicate twofold increase or decrease of expression as compared with the control group.

**Figure 2:**
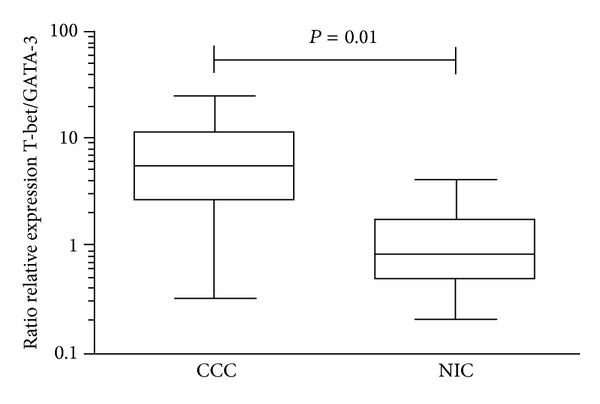
The ratio of mRNA encoding transcription factors T-bet and Gata-3 in myocardium. The ratio of relative expression of T-bet/GATA-3 in CCC and NIC group. The ratio of relative expression was plotted in comparison to N group and data were calculated with the 2^−ΔΔCt^ method, as described in Methods section.

We evaluated the expression of the transcription factors associated with the T_H_1, T_H_2, and T_H_17 effector T cell lineages. The expression of mRNA encoding the transcription factors* T-bet* and* GATA-3* was 10 and 2-fold higher in CCC samples than in NIC samples, respectively (*P* = 0.001 and *P* = 0.01, resp.; Figure 1). However, the expression of* ROR*γ*-*T mRNA, the master transcription factor for T_H_17 cells, was not significantly different in the myocardium of CCC patients when compared to heart of NIC patients and control individuals (Figure 1). The ratio of relative expression of* T-bet*/*GATA-3*, a putative index of T_H_1/T_H_2 imbalance [32], was significantly higher in the CCC than in the NIC group (Figure 2), indicating once again the skewed T_H_1/T_H_2 balance in CCC myocardium.

### 3.3. Hallmark T_H_1, T_H_2 and T_H_17 Cytokine Expression in CCC Patient Myocardial Tissue

**Figure 3:**
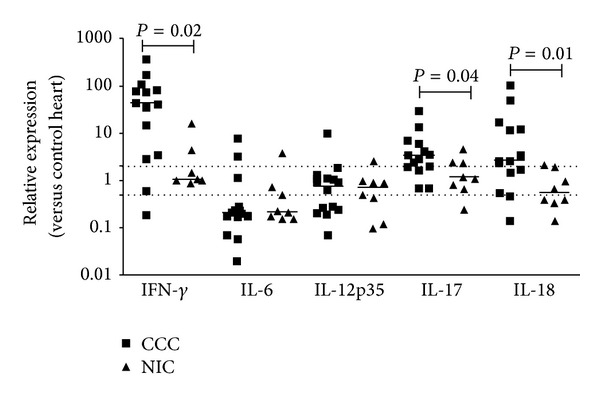
Myocardial expression of cytokine mRNA. Real-time qPCR analysis of mRNA expression in CCC and NIC myocardium. After normalization to GAPDH mRNA, relative increase was plotted in comparison to N group and data were calculated with the 2^−ΔΔCt^ method, as described in Methods section. The horizontal bar stands for the median. Dotted lines indicate twofold increase or decrease of expression as compared with the control group.

Given the evidence for the expression of* T-bet* and* GATA-3* mRNA in CCC myocardium, indicative of the presence of T_H_1 and T_H_2 cells, we also evaluated mRNA expression of hallmark T_H_1, T_H_2, and T_H_17 cytokines. Expression levels of* IFN*-*γ* and the proinflammatory and pro-T_H_1 cytokine* IL-18* were 42- and 3-fold higher in the heart tissue of CCC than NIC patients (*P* = 0.02 and *P* = 0.01, resp.; Figure 3). We observed a positive correlation between* T-bet *expression with that of* IFN*-*γ* and* IL-18*; significantly, mRNA expression of* T-bet* was also positively correlated with left ventricular diastolic diameter (LVDD), an index of global systolic ventricular dysfunction (Table 2). T_H_2 cytokines* IL-4*,* IL-5*, and* IL-13* were undetectable in all samples, while* IL-17* expression was 3-fold higher among CCC than NIC samples (*P* = 0.04) (Figure 3 and data not shown). However, expression of other proinflammatory cytokines such as* IL-1* *β* ,* IL-12p40*,* IL23p19*, and* IL-27*, which also has regulatory functions [35], was undetectable in all samples tested (data not shown), while expression of* IL-6* and* IL-12p35* both in the CCC and NIC groups was similar to that found in control samples (Figure 3).

### 3.4. Expression of Molecules Associated with Regulatory T Cell Function on Heart Tissue from CCC Patients

**Figure 4:**
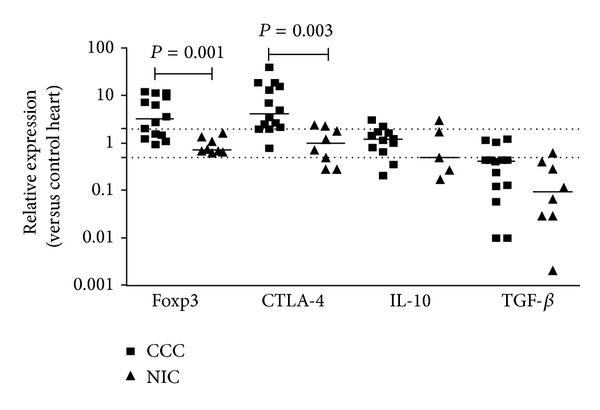
Expression of Foxp3, CTLA-4, IL-10, and TGF-*β* in myocardium. Real-time qPCR analysis of mRNA expression in CCC and NIC myocardium. After normalization to GAPDH mRNA, relative increase was plotted in comparison to N group and data were calculated with the 2^−ΔΔCt^ method, as described in Methods section. The horizontal bar stands for the median. Dotted lines indicate twofold increase or decrease of expression as compared with the control group.

We next analyzed the expression of genes associated with regulatory T cell function in myocardial samples from the three groups. mRNA expression of* FoxP3* and* CTLA-4* was 3- and 5-fold higher in the heart tissue of CCC than in NIC patients, respectively (*P* = 0.001 and *P* = 0.003, resp.; Figure 4). On the other hand, there was no significant difference in the expression of* IL-10* and* TGF*-*β* in myocardial samples of CCC patients when compared to those of NIC patients and control individuals (Figure 4). We found a significant (*r* = 0.77, *P* = 0.001) positive correlation between the mRNA expression of* FoxP3* and* CTLA-4* (Table 2), which is consistent with coexpression in the same cell population. Expression of genes associated with T_H_1 cells, such as* IFN*-*γ* ,* T-bet*, and* IL-18*, was positively correlated with the Treg-associated molecules* FoxP3* and* CTLA-4*;* T-bet* expression correlated highly significantly with* CTLA-4* (*r* = 0.90, *P* = 0.001) (Table 2).

## 4. Discussion 

We report that CCC myocardial tissue displays significantly increased expression of mRNA encoding* IFN*-*γ* and* T-bet*, with less prominent increase in expression of* IL-17*,* GATA-3*,* FoxP3*, and* CTLA-4*. Among proinflammatory cytokines only* IL-18*, but not* IL1* *β* ,* IL-6*,* IL-12*,* IL-23*, and* IL-27*, displayed increased expression in CCC heart tissue. mRNA expression of the T_H_2 cytokines* IL-4*,* IL-5*, and* IL-13*, and cytokines associated with regulatory T cells, such as* IL-10* and* TGF*-*β*, was either similar to controls or undetectable. T_H_1-associated genes such as* T-bet*,* IFN*-*γ* , and* IL-18* expression levels were found to correlate among themselves, as well as with* FoxP3*,* CTLA-4*, and, in the case of T-bet, with ventricular dilation. Transcription factor and cytokine expression patterns are consistent with a predominant T_H_1-type inflammatory infiltrate, with antagonized T_H_2 cells and a proportionately smaller FoxP3^+^CTLA-4^+^ Treg cell population which fails to completely suppress IFN-*γ* production and T_H_1 inflammation in CCC myocardium. The correlation of T-bet and ventricular dysfunction further points out the role of inflammatory T_H_1 responses in pathological myocardial hypertrophy/remodeling leading to disease progression.

The finding that the expression of* T-bet* is significantly upregulated in CCC myocardial tissue corroborates the predominance of T_H_1-type of heart-infiltrating T cells in the CCC myocardium. The finding that the median* IFN*-*γ* mRNA expression was over 40-fold upregulated in CCC myocardial tissue is in line with previous studies of heart-infiltrating T cell lines and immunohistochemical studies [14, 25, 36]. Our group has recently shown that expression of IFN-*γ*- inducible chemokines* CXCL9* and* CXCL10* may be directly involved in the recruitment of large numbers of CCR5^+^ and CXCR3^+^T_H_1-type T cells to CCC myocardium [24, 26], suggesting that the local production of IFN-*γ* and IFN-*γ* inducible chemokines leads to the recruitment of effector T_H_1-type T cells into heart tissue. The correlations between the T_H_1-associated genes* T-bet*,* IFN*-*γ* , and* IL-18* and* CCR5*,* CXCR3*, and their IFN-*γ*-dependent chemokine ligands were described previously in the same sample set (Table S2) [26]. Although we measured static mRNA levels in a single time point, this can be a sign of a positive feedback loop. Increased numbers of cells capable of local production of IFN-*γ* and also IFN-*γ*-dependent chemoattractant molecules may result in the migration of additional CCR5+, CXCR3+, IFN-*γ* producing T1-type T cells. The correlation between* T-bet* expression levels and the left ventricular diastolic diameter, an index of ventricular dilation and disease severity, is consistent with the idea that the T_H_1-type T cell compartment is a determinant factor in CCC progression. In support of this idea, associations between the intensity of the inflammatory infiltrate and disease progression have been previously described in Chagas disease patients [8] and in the chronic Syrian hamster model of CCC where the number of mononuclear cells also correlated with ventricular dilation (ECN and JK, unpublished data). This is further corroborated by the positive correlation between the intensity of lymphocytic myocarditis and fibrosis [26] and may be the pathogenetic translation of the ability of IFN*γ* to directly induce ANF expression in cardiomyocytes [24], the first step in the pathological hypertrophy pathway. Accordingly, a recent report has described that IFN-*γ* overexpressing transgenic mice develop mononuclear cell myocarditis, culminating in dilated cardiomyopathy [37].

The modest expression of* GATA-3*, together with the observed lack of expression of* IL-4*,* IL-5*, and* IL-13*, hallmark effector T_H_2 cytokines, suggests that T_H_2 cells may be relatively rare in the CCC myocardial infiltrate and failing to produce T_H_2 cytokines, thus being nonfunctional possibly due to antagonism by IFN-*γ* [38]. Our findings are in contrast with previous immunohistochemistry studies that, in spite of showing a majority of mononuclear cells staining with anti-IFN-*γ*, disclose a minority of mononuclear cells producing IL-4 in CCC myocardium [25, 30] but are in agreement with a previous study with T cell lines derived from CCC myocardium [13]. At any event, STAT4 mRNA was overexpressed in CCC patients with heart failure as compared with STAT6 levels in patients with presence or absence of heart failure [30], a further indication of T_H_1 signaling [24]. The correlation found between* GATA-3* expression and CCR4 (Table S2) may suggest that infiltrating T_H_2 cells effectively possess such a phenotype.

In the absence of* ROR*γ*-T* expression, the finding of low-grade expression of* IL-17* suggests that there may be little or no differentiated T_H_17 cells in CCC heart tissue. At any event, the correlation found between* IL-17* expression and* CCR4* (Table S2) may suggest that such putative infiltrating T_H_17 cells effectively possess this phenotype. This may be in concert with the recent finding that CCC patients with low ejection fraction similar to the ones examined here had lower IL17^+^ T cells in their PBMC than CCC patients without ventricular dysfunction [23].

Our finding of a modest increase in the mRNA expression of FoxP3 and CTLA-4, with no significant modulation of TGF-*β* and IL-10 expression, is in line with previous studies showing that FoxP3+ cells are significantly less abundant in myocardial sections from CCC than in ASY patients or noninfected individuals, suggesting that reduced numbers of Treg cells could be one important cause for the prevalent T_H_1 response in CCC heart tissue [29]. Araujo et al. [21] have previously shown that PBMC from CCC patients displayed increased numbers of CD4^+^CD25^high^Foxp3^+^CTLA-4^+^ T cells and decreased numbers of CD4^+^CD25^high^IL-10^+^ T cells, as compared to ASY patients, consistent with our findings in regulatory T cell molecules in CCC heart tissue [22]. Recently, CTLA-4 was found to be expressed in mononuclear cells infiltrating heart tissue sections from chronically infected subjects with severe myocarditis [39]. The finding that expression of FoxP3 and CTLA-4 displayed positive correlations with T_H_1 chemokine receptors CCR5 and CXCR3 and their ligands, along with T-bet, IFN-*γ*, and IL-18 (Table S2), is in line with previous findings and indicates for the first time that the FoxP3^+^ CTLA4^+^ Treg compartment bears a relationship with the T_H_1 infiltrate. However, in case the Treg compartment was effectively controlling the T_H_1 infiltrate in at least some samples, one would expect to find a negative correlation between markers of the two T cell populations. Data thus suggest that a proportional but comparatively smaller or less functional FoxP3^+^ CTLA-4^+^ Treg compartment, possibly also bearing chemokine receptors CCR5 and/or CXCR3 [38], migrated to CCC heart tissue in a partially failed attempt to control T_H_1-driven inflammation. However, since both FoxP3 and CTLA-4 can be transiently expressed in activated human T cells, we cannot formally exclude that the increased expression was merely due to the presence of activated T cells belonging to other functional subsets [40, 41]. Our findings of lack of upregulation of* TGF*-*β* in situ are in apparent contrast with the immunohistochemical study by Araúijo-Jorge et al. [33] who have identified a low number of TGF-*β* ^+^ mononuclear cells infiltrating CCC myocardium. However, that report failed to show values from healthy control tissue samples, so it is not possible to assess whether the detected values were above baseline. A recent report showed that circulating TGF-*β*1 could be detected in CCC serum samples [42] which could be the source of activation of the TGF-*β*1 signaling pathway in CCC myocardium [28].

The selective increase of* IL-18* in the absence of any other proinflammatory cytokine in CCC myocardium is intriguing, since most proinflammatory cytokines are produced in response to shared stimuli, like Toll-like receptor ligands and IFN-*γ* [43]. The longer half-life of the* IL-18* mRNA [44] could partially explain our findings. The positive correlation between the mRNA expression of* IL-18* and* IFN*-*γ* is consistent with the described positive feedback loop between the two cytokines [45]. IL-18 has been reported to induce ANP gene expression and hypertrophy in cardiomyocytes, as previously described for IFN-*γ*, TNF-*α*, IL-1*β*, and CCL2 [24, 46]. IL-18 also induces fibroblast expression of fibronectin, a prominent extracellular matrix protein [47], a mechanism possibly involved in myocardial fibrosis.

Since all our CCC myocardium samples came from clinically similar end-stage patients submitted to transplantation, it could be argued that possessing a more or less intense expression of T-bet, a T_H_1-associated expression profile or even a more significant inflammatory infiltrate by itself, may not be relevant for the progression of CCC. However, CCC is not a monogenic disease, and it is likely that the progression to overt inflammatory dilated cardiomyopathy may result from the combined effect and inadequate counterregulation of relevant genes and environmental factors. Polymorphisms in multiple innate immunity/inflammatory genes have been found to associate with risk for developing CCC (reviewed in [5, 11]). In addition to interference by other genes, differential myocardial resilience, including responses to hypertrophic/fibrogenic factors occurring in CCC heart tissue (*IL1* *β* ,* TNF*-*α* ,* IFN*-*γ* ,* IL18*,* CCL2*,and* CCL21*)(reviewed in [5]), could explain why these few patients presenting less intense inflammation and a lower expression of T_H_1 cytokines can nevertheless develop end-stage cardiomyopathy. Our group has recently observed that polymorphisms in the promoter region that bind to transcription factors of the cardiac actin gene, a cardiomyocyte gene associated with muscle contraction and resilience, whose dysfunction or altered expression levels lead to cardiomyocyte malfunction and apoptosis [48] associate with CCC development [49]. In the Syrian hamster model of chronic Chagas disease cardiomyopathy, although the intensity of chronic inflammation correlated with ventricular dilation, intensity of myocarditis was similar in hamsters dying from chronic* T. cruzi*-induced dilated cardiomyopathy and survivors euthanized 11 months after infection [5], suggesting the existence of additional factors related to disease progression or death from CCC.

It is likely that the interplay between the Treg and T_H_1-type T cell populations is key towards the control of myocardial inflammation in chronic Chagas disease. Our findings suggest that the myocarditis in the chronic cardiac form of Chagas disease is related to a strong T_H_1 response in most cases, associated with a balanced regulatory T cell response and an antagonized T_H_2 response. Our results are consistent with the hypothesis that a putative FoxP3^+^ and CTLA-4^+^ Treg heart-infiltrating T cell population fails to control the exacerbated IFN-*γ* production by T_H_1-type T cells in the majority of end-stage CCC cases.

## 5. Conclusion

The T_H_1-type T cell-rich mononuclear infiltrate plays a major role in the development and progression of chronic CCC. We found increased expression of T_H_1-associated genes in CCC myocardial tissue, with minor upregulation, similar or even undetectable levels of mRNAs encoding associated T_H_2, T_H_17 and Treg associated genes. Our results show a limited role of T_H_2-type T cells, and are consistent with the hypothesis that a putative FoxP3^+^ and CTLA4^+^ Treg heart-infiltrating T cell population fails to control the exacerbated IFN-*γ* production by T_H_1-type T cells in the majority of end-stage CCC cases.

## Supplementary Material

Supplemental table 1 (Table S1) depicts sequences and features of PCR primers used in this study. Supplemental table 2 (Table S2) depicts correlation tests between expression of genes in myocardial samples tested in this study (cytokines and transcription factors) to those evaluated in Nogueira et al. 2012 (Ref. 26) – chemokines and chemokine receptors. Both studies were made in the same myocardial sample set.

## Figures and Tables

**Table 1:** Characteristics of patients and control heart donors whose samples were used in this study.

	CCC	NIC	N
*n*	14	8	6
Age	47.2 ± 14.6	53.3 ± 7.5	32.2 ± 12.8
Sex (M/F)	5/9	0/9	0/6
EF	26.50 ± 8.96	22.73 ± 6.28	ND
Fibrosis	Moderate to intense	Moderate to intense	0
LVDD	71.64 ± 7.48	75.86 ± 15.84	ND
Hypertrophy	Yes	Yes	No
Myocarditis	Moderate to intense	Absent	0

Age (years); M (male); F (female); CCC (chronic Chagas cardiomyopathy); NIC (noninflammatory cardiomyopathy). Normal heart donors (N) were subject to ventilator and vasoactive drugs and had been under life support for an average of 48 hours. Characterization of the samples as myocarditis, fibrosis, and hypertrophy; reference values for the presence of myocarditis and fibrosis: absent; slight; moderate; intense; hypertrophy: Y (yes), N (no). ND (not done); EF (left ventricular ejection fraction) ≥ 55%; LVDD (left ventricle diastolic diameter); reference value: diameter 39–55 mm.

**Table 2:** Correlation of mRNA expression of T cell lineage-associated molecules against each other and versus LVDD on heart tissue from CCC patients using Spearman's rank correlation.

mRNA expression	*P*	*r*
T-bet versus LVDD	0.043	0.546
T-bet versus Foxp3	0.047	0.538
T-bet versus CTLA-4	0.0001	0.903
IFN-*γ* versus Foxp3	0.004	0.714
IFN-*γ* versus CTLA-4	0.004	0.710
IL-18 versus T-bet	0.045	0.543
IL-18 versus IFN-*γ*	0.002	0.749
IL-18 versus Foxp3	0.009	0.670
IL-18 versus CTLA-4	0.007	0.648
Foxp3 versus CTLA-4	0.001	0.771
