# Supplementary material for: Myocardial Gene Expression of T-bet, GATA-3, Ror-γt, FoxP3, and Hallmark Cytokines in Chronic Chagas Disease Cardiomyopathy: An Essentially Unopposed TH1-Type Response
Source: Mediators Inflamm. 2014 Jul 24;2014:914326. doi: 10.1155/2014/914326 (PMC4134835; doi:10.1155/2014/914326)
Supplement: Supplementary file 1 — Supplemental table 1 (Table S1) depicts sequences and features of PCR primers used in this study. Supplemental table 2 (Table S2) depicts correlation tests between expression of genes in myocardial samples tested in this study (cytokines and transcription factors) to those evaluated in Nogueira et al. 2012 (Ref. 26) – chemokines and chemokine receptors. Both studies were made in the same myocardial sample set. [file 914326.f1.pdf]

Table S1. Characteristics of the primers.

Gene Name	Gene bank accession number	Sequence 5'→ 3'	Amplicon (pb)	Tm Amplicon (ºC)
IL-1β	M15330	AGGATATGGAGCAACAAGTGGTG<br>ATTCTTTTCCTTGAGGCCCAAG	98	80
IL-4	M13982	TCCGATTCCTGAAACGGCT<br>TCTGGTTGGCTTCCTTCACAG	81	83
IL-5	NM_000879	CTCATCGAACTCTGCTGATAGCC<br>CAGTGTGCCTATTCCCTGAAAGTA	110	75
IL-6	M14584	TCAGCCCTGAGAAAGGAGACATG<br>GCATCCATCTTTTTCAGCCATCT	111	77
IL-12p35	NM_000882	GCAAAGCTTCTGATGGATCCTAAG<br>TTGAAATTCAGGGCCTGCAT	92	77
IL-12p40	NM_002187	TGTCAAAAGCAGCAGAGGCTCT<br>CTTGTTGTCCCCTCTGACTCTCTC	91	82
IL-13	X69079	ACCTGACAGCTGGCATGTACTG<br>AGAATCCGCTCAGCATCCTCT	99	82
IL-17	NM_002190	CAATGACCTGGAAATACCCAA<br>TGAAGGCATGTGAAATCGAGA	52	71
IL-18	D49950	GCATCAACTTTGTGGCAATGAAA<br>AAGCTTGCCAAAGTAATCTGACTCC	95	73
IL-23	NM_016584	GGACAACAGTCAGTTCTGCTTGC<br>AGGCTCCCCTGTGAAAATATCC	91	78
IL-27	NM_145659	CCCTGATGTTTCCCTGACCTTC<br>CAGGGCATGGAAGGCTGAA	97	85
TGF-β	M38449	GGTGGAAACCCACAACGAAAT<br>TCTCGGAGCTCTGATGTGTTGA	85	75
Foxp3	NM_014009	GAGAAGGGCAGGGCACAAT<br>TGGGCCTGCATGGCAC101	101	83
T-bet	NM_013351	GGATGCGCCAGGAAGTTTCA<br>GACTGGAGCACAATCATCTGGG	91	79
GATA-3	NM_001002295	AGCACAGAAGGCAGGGAGTGT<br>TGATAGAGCCCGCAGGCG	104	85
Rorγ-T	Anm_005060	GGCAAATACGGTGGCATGG<br>AAGGCACTTAGGGAGTGGGAGA	92	82
CTLA4	NM_005214	CATGGACACGGGACTCTACATCT<br>GGCACGGTTCTGGATCAATTAC	110	81
IFN-γ	NM_000619.2	GTGTGGAGACCATCAAGGAAGACA<br>TTGGACATTCAAGTCAGTTACC	110	75
GAPDH	NM_002046	TGGTCTCCTCTGACTTCA<br>AGCCAAATTCGTTGTCAT	117	82

Table S2. Correlation of mRNA expression of T cell lineage-associated molecules against chemokines and their ligands on heart tissue from CCC patients using Sperman rank correlation.

mRNA expression	p	r
T-bet vs. CXCL9	0.006	0.692
T-bet vs. CXCL10	0.014	0.640
T-bet vs. CXCR3	0.009	0.666
T-bet vs. CCL4	0.002	0.745
T-bet vs. CCL5	0.015	0.635
IFN-γ vs. CXCL9	0.002	0.741
IFN-γ vs. CXCL10	0.032	0.574
IFN-γ vs. CXCR3	0.015	0.635
IFN-γ vs. CCL5	0.032	0.574
IFN-γ vs. CCR5	0.011	0.653
IL-18 vs. CXCL9	0.001	0.771
IL-18 vs. CXCL10	0.039	0.556
IL-18 vs. CXCR3	0.0001	0.908
IL-18 vs. CCL5	0.014	0.640
IL-18 vs. CCR5	0.008	0.675
IL-17 vs. CCR4	0.009	0.671
FoxP3 vs. CXCL9	0.007	0.684
FoxP3 vs. CXCL10	0.016	0.631
FoxP3 vs. CXCR3	0.019	0.618
CTLA-4 vs. CXCL9	0.002	0.741
CTLA-4 vs. CXCL10	0.002	0.758
CTLA-4 vs. CXCR3	0.005	0.701
CTLA-4 vs. CCL4	0.001	0.789
CTLA-4 vs. CCL5	0.011	0.653
CTLA-4 vs. CCR5	0.022	0.604
GATA-3 vs. CCR4	0.043	0.547
